# How Do Art Skills Influence Visual Search? – Eye Movements Analyzed With Hidden Markov Models

**DOI:** 10.3389/fpsyg.2021.594248

**Published:** 2021-01-28

**Authors:** Miles Tallon, Mark W. Greenlee, Ernst Wagner, Katrin Rakoczy, Ulrich Frick

**Affiliations:** ^1^Department of Experimental Psychology, University of Regensburg, Regensburg, Germany; ^2^HSD Research Centre Cologne, HSD University of Applied Sciences, Cologne, Germany; ^3^Academy of Fine Arts Munich, Munich, Germany; ^4^DIPF Leibniz Institute for Research and Information in Education, Frankfurt am Main, Germany

**Keywords:** visual literacy, assessment, fixation sequence, hidden markov model, eye tracking data, visual search task, latent profile analysis

## Abstract

The results of two experiments are analyzed to find out how artistic expertise influences visual search. Experiment I comprised survey data of 1,065 students on self-reported visual memory skills and their ability to find three targets in four images of artwork. Experiment II comprised eye movement data of 50 Visual Literacy (VL) experts and non-experts whose eye movements during visual search were analyzed for nine images of artwork as an external validation of the assessment tasks performed in Sample I. No time constraint was set for completion of the visual search task. A latent profile analysis revealed four typical solution patterns for the students in Sample I, including a mainstream group, a group that completes easy images fast and difficult images slowly, a fast and erroneous group, and a slow working student group, depending on task completion time and on the probability of finding all three targets. Eidetic memory, performance in art education and visual imagination as self-reported visual skills have significant impact on latent class membership probability. We present a hidden Markov model (HMM) approach to uncover underlying regions of attraction that result from visual search eye-movement behavior in Experiment II. VL experts and non-experts did not significantly differ in task time and number of targets found but they did differ in their visual search process: compared to non-experts, experts showed greater precision in fixating specific prime and target regions, assessed through hidden state fixation overlap. Exploratory analysis of HMMs revealed differences between experts and non-experts in image locations of attraction (HMM states). Experts seem to focus their attention on smaller image parts whereas non-experts used wider parts of the image during their search. Differences between experts and non-experts depend on the relative saliency of targets embedded in images. HMMs can determine the effect of expertise on exploratory eye movements executed during visual search tasks. Further research on HMMs and art expertise is required to confirm exploratory results.

## Introduction

Visual perception is an active process of constructing meaningful information from external visual stimuli based both on neurobiological capacities (i.e., laws of perception) and individual learning history (skill training, memory). Perceptual psychology describes the cognitive mechanisms employed to transform visual stimuli into information. The comparison of experts’ and non-experts’ processing during a challenging visual task can be used to decipher these cognitive mechanisms. In a broad sense, visual expertise has been studied in medicine (medical imaging), engineering (surveillance of technical processes) or education (learning behavior) and has been defined as a domain-specific adaptation to the requirements of a visually challenging task (Gegenfurtner and van Merriënboer, 2017), which has been coined Visual Literacy (VL). More recently, mostly from authors in the context of aesthetics and fine arts, this concept has also been referred to as visual competency (Schönau and Kárpáti, 2019). Other authors (Avgerinou and Pettersson, 2011; Wagner and Schönau, 2016) used the term VL, which they described as the ability to inspect and understand images and express oneself through visual media.

Psychological models of visual expertise have focused on three major theories (Gegenfurtner et al., 2011; Brams et al., 2019): (1) the long term working memory theory (Ericsson and Kintsch, 1995) suggests that experts can retrieve more visual information from long term working memory than novices do, (2) the information reduction hypothesis (Haider and Frensch, 1996, 1999) proposes that experts selectively focus on important visual image parts relevant for the task and ignore irrelevant stimuli, and (3) the holistic model of image perception (Kundel et al., 2007), which states that experts gain more visual information from global and para-foveal regions, effectively allowing a broader grasp of the image to guide their search. Recent studies find evidence in support of the information-reduction hypothesis as the most important skill developed in experts across most domains (Brams et al., 2019).

Restricting the discussion to fine art studies and art education, differing VL models have been proposed (Kędra, 2018). One of the broadest conceptual models (ENViL-model, see Wagner and Schönau, 2016) divides VL into as many as 16 subdomains, which include “value,” “envision,” “experiment,” or “aesthetic experience.” Many of these domains show considerable overlap. As this model was not generated by psychometricians but by art educators, this model is strictly phenomenological and has not been empirically tested. Nevertheless, it has received great attention from applied art education theory (e.g., Groenendijk et al., 2018). One subdomain, “analyzing,” has been described as the ability to attentively and accurately focus on visual stimuli and to identify characteristics of images (Wagner and Schönau, 2016, p. 70) and therefore should be crucial in visual search tasks. The “analyzing” ability is directly associated with the information-reduction hypotheses, where experts find important visual information by focusing on important image features and ignoring irrelevant features. Experts’ continuous engagement in art and imagery plausibly should impact on how cognitive processes differ between VL experts and non-experts. VL experts thus may serve as best-practice examples to describe effective cognitive strategies in detecting details in images of artwork.

In a visual search experiment the participant is asked to look for a target among distractors (Wolfe et al., 2003; Wolfe, 2010). A visual search experiment might be analyzed by either evaluating the correct solution or by recording task-solving behavior (e.g., reaction times and eye movements). Thus, visual search paradigms are oftentimes used to investigate differences of experts and novices with respect to the participant’s speed or accuracy in locating targets, for example in medical image examination (Drew et al., 2013; Sheridan and Reingold, 2017; van der Gijp et al., 2017) or in sports (Vaeyens et al., 2007; Piras et al., 2014). The influence of *reading* literacy on visual search has been extensively studied (Ferretti et al., 2008; Franceschini et al., 2012; Olivers et al., 2013). Few studies have considered the influence of *visual* literacy and its effects on visual-search performance. Studies on artistic visual expertise (e.g., Vogt and Magnussen, 2007; Francuz et al., 2018) are typically not conducted with visual search tasks (finding targets among distractors) but, e.g., by judging abstract from realistic paintings or in the context of visual memory tasks. Expertise-related differences in target search have been mainly explored in domains other than the visual arts [e.g., medicine (Kundel et al., 2007) or sports (Vaeyens et al., 2007)]. To our knowledge, the use of artwork in a visual search task still remains fairly uncommon (e.g., Nodine et al., 1979).

Aesthetic appreciation and a general interest in the visual arts might amplify a person’s ability to identify specific details in images of artwork. With respect to art appreciation, five domains have been put forward: A (attraction), R (representation and realism), E (emotional expression) S (style and form), and I (interpretation), denoted as ARESI classification (van Meel-Jansen, 2006). Visual experts tend to show more appreciation for images rated high on the “style and form” and “realism” domain. Evidence from neuroaesthetic research revealed perceptual processing enhancement at behavioral and at a neurophysiological level when images are aesthetically appreciated (Sarasso et al., 2020). Art appreciation might facilitate visual search performance. Research on the aesthetic appreciation of art has differentiated between two modes of perception: pleasure and interest, which are conceptualized as partly overlapping, partly distinct functions of aesthetic appreciation (Graf and Landwehr, 2017). Whereas the free viewing of abstract paintings presumably favors the “pleasure” mode, a visual search task for a specific detail in paintings might require a more analytical way of regarding a piece of art (“interest” mode), depending on the painting style or content of the artwork. This might also elicit aesthetic appreciation. Thus, experts would operate more according to the “interest” pathway to aesthetic appreciation.

Studies of eye movement behavior often are regarded as a tool to link observed behavior to cognitive mechanisms (Hollingworth and Bahle, 2020). Previous eye-movement research has focused on domain-specific differences in visual expertise with respect to number of fixations and fixation duration (Gegenfurtner et al., 2011). Studies showed that professional art viewers were reported to exhibit greater saccadic amplitudes than novices, particularly when viewing abstract paintings (Zangemeister, 1995). Experts also tend to have more short fixation durations, i.e., direct their attention on specific areas of paintings (Ylitalo et al., 2016). Novices, when revisiting previously seen images, exhibit fewer and longer fixations (Vogt and Magnussen, 2007). Some studies used eye movement patterns to distinguish artists from laymen (Kolodziej et al., 2018). Other studies demonstrated differences between expert and novice artists in how they looked at particular works of art. Accordingly, experts differ from novices in the number of fixations and average fixation duration on specific parts of the image (Kolodziej et al., 2018). Interestingly, in a study on artists’ free viewing behavior of abstract paintings, Koide et al. (2015) report that expertise leads to fewer fixations on salient image regions. The authors suggest that the artists’ knowledge of art overrides stimulus-driven guidance of fixations, opening up the possibility of focusing their attention to less obvious image areas. Even though the transfer of VL ability across the art domain boundary remains uncertain, some studies have found differences in visual-spatial tasks depending on the person’s level of artistic expertise (Angelone et al., 2016; Chamberlain et al., 2019). In these studies, visual artists outperform novices through top-down control over attentional processes and fast and more precise visual encoding.

As previous studies pointed out differences in fixation duration or number of fixations, the time dependence of fixation sequences is rarely taken into account. However, the order of fixation sequences can be used to deepen our understanding of expertise-related differences in visual search. Eye-movements play an important role in visual search behavior, as they can indicate where and for how long people look at something, allowing researchers to model attention throughout the given task (Koochaki and Najafizadeh, 2018; Hollingworth and Bahle, 2020). Heatmaps (Bojko, 2009), for example, can be used to visualize fixation density (number of fixations) over time. However, sequences (spatial as well as chronological order) of eye movements (i.e., scanpaths) are often neglected in the analysis of saliency or fixation density (Le Meur and Baccino, 2013). The sequence of eye movements is crucial in understanding not only where, but in what order people direct their gaze and attention while inspecting images.

To account for sequence dependent effects in eye movements, we chose to compare the spatial coordinates of fixations (i.e., the series of numbered fixations by index) across VL expert and non-expert groups. We use Hidden Markov Models [HMM (Rabiner, 1989)] to analyze the fixation sequence to reveal latent image areas. Through HMM-analysis we can find and visualize hidden (i.e., not directly observable) attention states (for details see “Materials and Methods” section). Combining eye tracking analysis with statistical approaches such as HMM leads to further insight into factors underlying scanpaths during visual search (Borji and Itti, 2013; Coutrot et al., 2018; Koochaki and Najafizadeh, 2018; Ulutas et al., 2019). HMMs have been successfully used in combination with eye tracking data to parse fixations from saccades (Houpt et al., 2018), to depict processes underlying facial recognition (Chuk et al., 2014, 2019) or for information retrieval during reading (Simola et al., 2008). The feasibility of a latent state approach for the analysis of eye-tracking data has become increasingly popular in applied research areas such as marketing research (Netzer et al., 2017).

Which parts of the image are closely examined and in what order are they examined during visual search? Search effectiveness and the probability of finding pre-defined targets are only one aspect of visual search performance. The psychometric assessment of search time and number of correctly identified targets does not allow for a detailed understanding of the underlying search process. Using eye-tracking measurements search processes and differences in expertise strategies can be examined more closely.

The research reported here is part of a larger study (Rakoczy et al., 2019) on visual literacy (grant 01JK1606A). A test battery was constructed to assess various aspects of VL, which was administered to a large sample of high school students for psychometric evaluation. The reliable and valid measurement of VL could serve as a tool for quality management in educational settings and thus contribute to the improvement of art education. Two aspects are especially interesting for the study of VL: First, not much is known about self-reported artistic skills and VL performance in young students. What influence do self-reported visual skills have on search time and number of found targets of students? Self-confidence or interest in visual arts may facilitate engagement in artistic stimuli. Secondly, cognitive mechanisms employed to solve visually guided tasks are a necessary link to translate skill level measurement to didactic improvements and to sharpen the association between self-perceived visual competency and art teacher’s feedback. In addition to the psychometric evaluation of visually guided tasks, we compare VL experts and non-experts’ visual search process to uncover expertise-specific modes of search behavior. Eye-tracking is used to determine the external validity of the assessment items. Do VL experts differ from non-experts in search time and number of found targets? The comparison of both a student sample and a sample of VL-experts and novices can enhance our understanding of cognitive processes engaged in the visual tasks going beyond the measurement of performance (i.e., reaction times and hit rates; effectiveness) to also include information on order and precision of the search (efficiency).

This study addresses the following hypotheses:

H1a: VL experts identify more targets than students (Experiment 1).
H1b: VL experts are faster than students in finding the targets (Experiment 1).

H2a: VL experts identify more targets than non-experts (Experiment 2).
H2b: VL experts are faster than non-experts in finding the targets (Experiment 2).

To get insight into the search process we take a closer look at the participant’s eye-movements during the search: Do VL experts differ in spatial and/or chronological aspects of their scanpaths from non-experts? More specifically, do VL experts identify more and/or other meaningful regions of interest in images of artwork than non-experts do?

H3: VL experts show higher precision in target detection, i.e., exhibit eye movements to targets that differ from those of non-experts during visual search (Experiment 2).

The eye-tracking research questions are assessed through exploratory analysis with the help of HMM models to investigate differences between the search strategies of VL experts and novices. Differences can be interpreted as empirically derived hypotheses for future confirmatory analysis. As the use of HMM in the context of expertise research is relatively new, we give some examples of how this method can be advantageous over traditional eye-movement visualization and analysis.

## Materials and Methods

### Subjects

The data reported in this study was acquired as part of a larger research project on the assessment of Visual Literacy (Rakoczy et al., 2019) and is comprised of two samples: an assessment sample (Sample I) involving a large sample of high-school students and an eye-tracking sample (Sample II) consisting of VL experts and non-experts.

**FIGURE 1:**
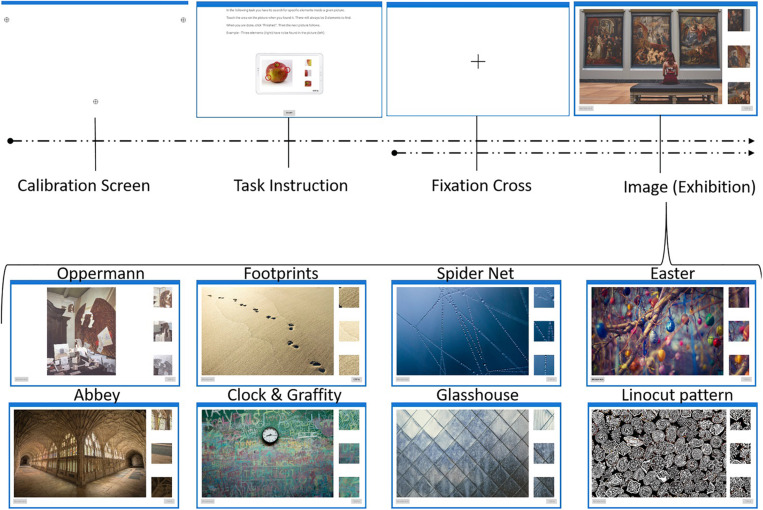
Procedure of experiment with nine images. Sample I only included the following images: Exhibition, Oppermann, Footprints and Clock and Graffiti. Sample II included all nine images. For more details see text.

Sample I comprised 1,065 high-school students from 52 classes (9th to 13th grade) of 29 schools in Germany of which 1,056 worked on the visual search task. Overall, 52% were female, the average age was 15.27 years (*SD* = 0.94). Schools were recruited in the federal states of Hessen, North-Rhine Westphalia, Schleswig-Holstein, and Rhineland Palatinate via leaflets, letters and personal recommendations. The test was conducted in regular classrooms. Up to 30 students were able to participate in the assessment simultaneously. The visual search task under investigation was one segment of a longer (duration: 45 min) study on the topic of VL including a sociodemographic questionnaire (age and gender) and questions regarding the topic of art and personal experience with art: “Do you regularly attend an art school or art workshops?” Scale from 1 (never) to 4 (multiple times a week), “Art is important for me personally,” “My parents are interested in art and artistic subjects,” “In our family, art is very important,” “We like to talk about art and artistic subjects in our family” on a scale from 1 (strongly disagree) to 4 (strongly agree); “How good are you at art theory (e.g., interpreting pictures, understanding art history)?” “How well do you perform in arts education generally?” from 1 (very bad) to 5 (very good) and questions including the grade in art class and self-reported skills: photographic memory (PM; “I have a ‘photographic memory”’), spatial orientation (SO; “When I see a photographed geometric object, I can imagine what it looks like from behind”), long-term memory (LM; “I can remember small details in pictures”), imagination (IM; “I can visualize things mentally”), and interest in visual puzzles (IP; “I like to solve picture puzzles”). These were reported on a scale from 1 (strongly disagree) to 4 (strongly agree). All answers were given via touchscreen input by the participants. School classes were offered a lump sum of 100€ as collective compensation. Sample I was presented four images included in the visual search task: “Exhibition,” “Oppermann,” “Footprints,” and “Clock and Graffiti” (see Figure 1).

Sample II comprised 52 participants, who were screened for eligibility as part of the eye-tracking study. Two participants were excluded from further analysis, one because of poor acuity and the other because of insufficient eye tracking quality. For another participant in the expert group the eye-tracker lost the tracking signal on two trials and therefore data from this participant were only included for the remaining trials (images 1–7). As there is currently no validated test available on the assessment of VL, experts and novices were screened based on their prior experience and interest in the visual arts. Participants in the expert group (*n* = 25) were either members of the European Network of Visual Literacy (ENVIL) or working in professions requiring a high visual competency (photographer, gallerist, art educator, art designer, art students, or self-employed artists). The non-expert group (*n* = 25) were adults from the clerical and academic staff of various educational settings not associated with academic or professional work in the visual arts. The participants’ ages ranged from 16 to 66 years (*mean* age = 29.08 years, *SD* = 12.55 years). Participants in Sample II were individually assessed in seminar or laboratory rooms (e.g., at the Academy of Fine Arts in Munich) or at expert’s working places. All participants had normal or corrected-to-normal vision. Student participants received 20€ each as compensation. Other participants, including experts in the expert group, who were generally interested in the topic of visual literacy and eye tracking, participated without any compensation.

### Stimuli

Subjects were required to identify three targets (details) on each of the four (Experiment I) or nine (Experiment II) subsequently presented images. Figure 1 illustrates the procedure of the experiment. Calibration screen and fixation cross was only visible for Experiment II.

In a short pre-study, four untrained VL experts independently rated each of the photographs in our sample of images with the ARESI classification (van Meel-Jansen, 2006) on a scale from 1 (feature not present) to 7 (highly prominent feature). Ratings reached a reasonable mean interrater correlation of ICC = 0.50 (Shrout and Fleiss, 1979) and the following mean values: A = 4.8, R = 5.1, E = 3.6, S = 5.1, I = 4.4. Thus, images can be regarded as of satisfactory aesthetic value with above average rating on realism (R) and style and form (S) and not representing outliers on one of the five aesthetic domains.

**FIGURE 2:**
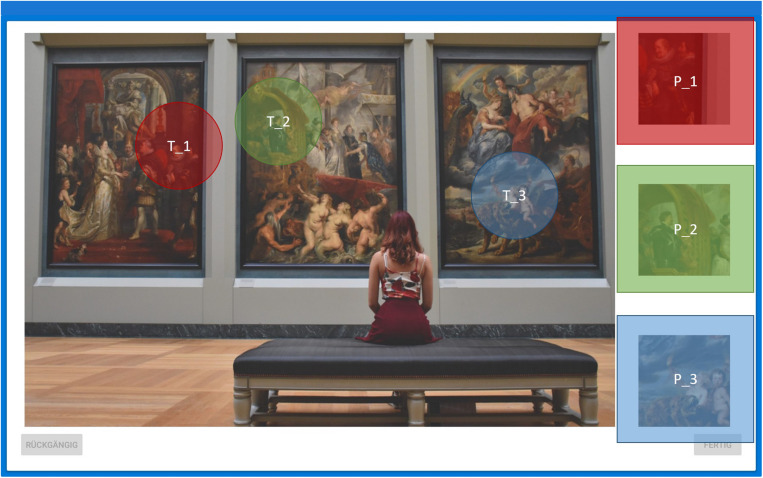
Example image with pre-defined AOIs. Primes (P_1–P_3) on the right-hand side and targets (T_1–T3) of the image are correspondingly shaded in color. Note that neither the colored shading nor the labeling of AOIs was presented to the subjects during the experiments. See Supplementary Material for all nine images.

Each image had three primes positioned on the right-hand side (P_1, P_2, and P_3). The primes represented details to be found as targets on the left (T_1 to T_3). Figure 2 shows prime and associated targets as pre-defined AOIs (see E-Appendix for all nine images). Image areas not covered by any AOI are defined as white space (WS). Once the subject found (or thought to have found) a target they touched the region on the screen to indicate the corresponding position of the given target. A red circle of 50-pixel radius appeared at each touching point to indicate that input was registered. Targets were counted as identified when they were touched within a 50-pixel radius around the center of each target region. All participants were assessed on Android A6 Tablets with 10.1-inch screen size. Tasks were constructed explicitly for the study (Andrews et al., 2018). There was no time constraint during the task. The participant ended each trial by pressing the “Done”-button.

In Sample II eye movements were recorded with SMI eye tracking glasses (SMI ETG 2w Analysis Pro). The glasses were positioned and strapped tightly onto the subject’s head, which they could freely move during task completion. Participants were seated 50–80 cm away from the tablet screen. Eye movements where calibrated with a 3-point calibration. All eye tracking data were recorded at 60 Hz. Saccades and fixations (as well as blinks) were recorded binocularly. Before each image was presented, a fixation cross was displayed for 2 s. Subjects were free to search the targets in any order and received no further feedback during the trial (on number of correct targets found).

The session started with a task instruction (in German):

“In the following task you have to search for specific details inside a given picture. Touch the area on the picture where you found it. There will always be 3 details to find. When you are done, click ‘Done’. Then the next picture follows.”

Eye tracking data analysis was conducted with SMI BeGaze^TM^ version 3.7. Fixations for each image were mapped onto corresponding reference images using SMI fixation-by-fixation semantic gaze mapping (Vansteenkiste et al., 2015). Each reference image was divided into three prime and three target AOIs (see Figure 2). The following eye movement variables were analyzed: the spatial coordinates of each fixation, the fixation sequence and the fixation duration in milliseconds. Due to the explorative nature of this study, no measures against inflation of type I error were undertaken, as statistical tests were not regarded as confirmatory analyses.

The study was conducted according to the guidelines for human research outlined by the Declaration of Helsinki and was approved by the Ethics Committee of Research of the Leibniz Institute for Research and Information in Education, Frankfurt am Main (DIPF, 01JK1606A). All subjects and their legal representatives, respectively, had given written informed consent prior to participation.

### Latent Profile Analysis

**FIGURE 3:**
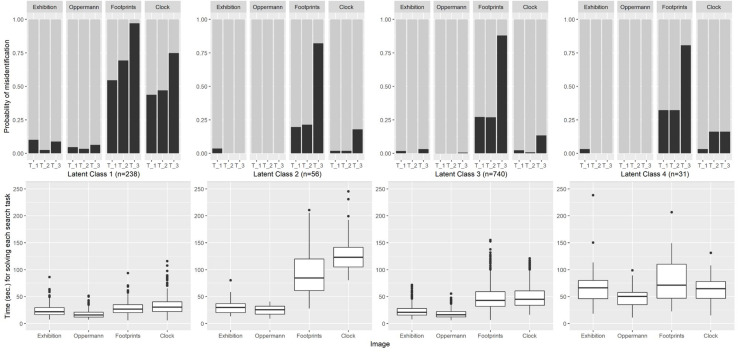
Probability of target misidentification for each latent class on each image **(Upper part)** and time for solving each search task on each image **(Lower part)**. Latent class profiles are depicted from left to right: LC1 “fast and erroneous” (*n* = 238), LC2 “easy images fast, difficult images slowly” (*n* = 56), LC3 “Mainstream” (*n* = 740), and LC4 “slow working” (*n* = 31).

Students’ responses to the images presented were recorded as a vector of 4 (images) times 3 (details to be identified) = 12 dichotomous variables (target correctly identified or not?) and 4 continuous variables (time in sec. to solve all three search tasks per image). Individual response patterns were grouped into latent classes of similar response patterns by means of a Latent Profile Analysis (see Ferguson et al., 2020), for statistical model and a practical application). Models between 2 and 6 latent classes were estimated using MPLUS 8.4 software. The decision to interpret four latent classes (named LC1–LC4 in Figure 3) as the final solution was based on the progression in the BIC fit indices (sharp decline after 4 classes) and the Lo-Mendell-Rubin test of significant improvements in model fit (*p* = 0.6707 for a five class solution). Latent class analysis (and the generalization of latent profile analysis) results in class membership probabilities for each individual to each of the estimated classes. Individual students were manifestly classified into latent classes according to their modal class membership probability. This categorical variable (reflecting four qualitatively differing solution patterns) then was used as dependent variable, which was regressed on by a list of demographic (gender and age) variables and self-reported skills (mentioned above under “subjects”). To arrive at a parsimonious multinomial logistic regression model for group membership, a stepwise selection of predictor variables was applied, which resulted in three significant predictors (Table 2). Results are presented as Odds-Ratios per scale point of three self-ratings of students’ visual performance skills for three of the latent classes as compared to the largest (“mainstream”) group.

### Hidden Markov Model

Hidden Markov models (HMMs) represent efficient and flexible modeling tools for data that include temporal constraints and spatial variability such as the sequences of eye movements. The intuitive idea behind a Markov Model or a Markov chain is that in series of events where each probability of something happening depends only on what happened right before it. For eye movements, we can look at the fixation sequence and classify each fixation to their most likely state (data-driven Area of Interest) depending on the previous fixation. The HMM divides the image into multiple data-driven AOIs which we can call Markov *states*. Each time a new fixation arises in our fixation sequence, we can give the fixation a certain probability of belonging to the same AOI or switching to any other one. This probability is called the *transition probability*. The transition probability is conditional to the previous fixation observed. Combining the probabilities for each state gives us a *transition matrix*. In the case of *hidden* Markov models the states are not directly observed. Only the observation sequence (the fixation sequence) is known.

A HMM comprises three components: the initial state distribution (in what states participants start in), the state transition probability distribution (how likely it is to transition from one state to another), and the observation probability distribution (how likely an observation is produced by any given state, i.e., how likely a fixation is linked to any given AOI). Again, each HMM state in this study represents a location on the image participants fixated while inspecting that area. The transitions between each hidden state (image area) can be placed into a transition probability matrix, describing the probability of switching between each hidden state.

A Hidden Markov model can be defined as

λ=A,B,π

where λ is a triplet comprising the model matrices. *A* is the state transition probability distribution of state *j* following state *i*. *B* is the observation (emission) probability distribution of observation k from the state *j*. π is the initial state distribution: π = *{*π*i}, 1* ≤ *i* ≤ *N.*

See (Rabiner, 1989) for an introduction to HMM and (Coutrot et al., 2018) and (Boccignone, 2019) for HMM applied to eye movement data.

Hidden Markov models were estimated using the depmixS4 package (Visser and Speekenbrink, 2010) with the software R. Spatial variability can be modeled through the output distribution of an HMM and temporal variability through the HMM transition parameters. In the case of eye-tracking data, each transition can be interpreted as an outgoing saccade from one data-driven AOI to another. The HMMs were formulated based on the spatial coordinates of each fixation. No additional constraints were put on the models’ parameter matrices. We estimated HMMs for each expertise group (experts vs. non-experts) on each of the nine images.

Each HMM was estimated from two to 14 states. Selection of model class (number of states for each image and group) was achieved by Bayesian Information Criteria (BIC, see Vrieze, 2012). If there was a discontinuous progression of the log-likelihood when incorporating a new state to the model, alternative seeds to determine randomly chosen starting values were used to avoid local optima.

To visualize the HMM, all fixations (emission points) were exhaustively and disjunctively classified to their best suited hidden Markov state and then drawn as 2D density maps (contour maps) onto the corresponding images. In order to analyze the precision of the visual search (H3) each pre-defined AOI (primes and targets) was linked to the hidden state with the highest number of fixations. Percentage of fixations inside pre-defined AOIs in each corresponding hidden state was used to determine precision. High fixation overlap of hidden state and AOI indicates higher precision while looking for prime and target regions.

**FIGURE 4:**
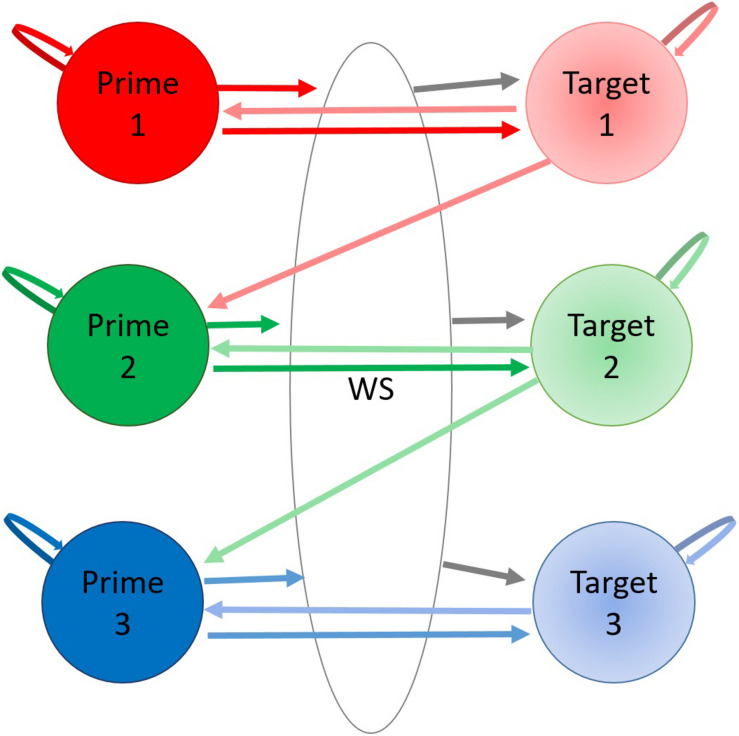
Hypothetical transition probability matrix for a theoretical HMM. In this simple arrangement each AOI represents a hidden state (Prime 1–3, Target 1–3, or White Space). Each hidden state has a certain probability of staying in that state or transitioning from one to another indicated by arrows. Note that the pairwise numbering is arbitrary as there was no instruction to search from top-to-bottom.

Figure 4 visualizes a hypothetical 7-state HMM of plausible transition probabilities between primes and targets.

## Results

### Speed and Precision of Search

Table 1 presents error rates for each target and task durations on each of the four images presented in Sample I and compares it to the corresponding results for Sample II. In Experiment I (Sample I) only images Exhibition, Oppermann, Footprints, and Clock and Graffiti were shown.

**TABLE 1:** Percent of correctly solved targets and mean time to solve each image in Sample I and Sample II.

Task		Total Sample I (*N* = 1056) Mean age = 15.27 years (*SD* = 0.94)		Sample II Experts (*N* = 25) Mean age = 34.36 years (*SD* = 14.69)		Sample II Non-experts (*N* = 25) Mean age = 23.80 years (*SD* = 6.92)	
			
Image	Target no.	Error Rate	Mean time in seconds (*SD*)	Error rate	Mean time in seconds (*SD*)	Error rate	Mean time in seconds (*SD*)
Exhibition	1	0.038	25.29 (15.14)	0.20	20.52 (6.58)	0.08	18.81 (6.02)
	2	0.006		0.00		0.00	
	3	0.041		0.04		0.04	
Oppermann	1	0.011	19.16 (10.10)	0.04	15.07 (6.77)	0.00	13.87 (7.15)
	2	0.008		0.00		0.04	
	3	0.018		0.00		0.00	
Footprints	1	0.331	47.21 (29.54)	0.32	44.06 (17.86)	0.20	48.73 (33.81)
	2	0.362		0.28		0.48	
	3	0.895		0.76		0.88	
Clock and Graffiti	1	0.115	50.39 (28.96)	0.12	55.18 (36.43)	0.12	47.63 (23.94)
	2	0.115		0.00		0.08	
	3	0.274		0.16		0.20	
Spider Net	1			0.56		0.68	
	2	Not applicable		0.08	41.25 (18.72)	0.08	35.41 (12.03)
	3			0.20		0.32	
Easter	1			0.12		0.16	
	2	Not applicable		0.08	37.29 (16.17)	0.24	33.73 (17.15)
	3			0.00		0.04	
Abbey	1			0.04		0.00	
	2	Not applicable		0.04	47.86 (25.00)	0.00	51.55 (20.00)
	3			0.20		0.60	
Glasshouse	1			0.12		0.28	
	2	Not applicable		0.40	60.12 (38.7)	0.40	57.90 (30.23)
	3			0.20		0.20	
Linocut Pattern	1			0.08		0.00	
	2	Not applicable		0.16	86.37 (40.4)	0.24	105.52 (62.69)
	3			0.04		0.08	

**TABLE 2:** Effect sizes (odds ratios) of self-reported art skills on latent profile classification.

Effect	Comparison group (referencemainstream”)	Odds ratio (per category)	95% confidence limits	
“I have a ‘photographic memory”’	LC4 “slow working”	0.961	0.632	1.463
	LC2 “easy images fast, difficult images slowly”	0.928	0.671	1.284
	LC1 “fast and erroneous”	0.659	0.554	0.785
“I can visualize things mentally”	LC4 “slow working”	0.991	0.659	1.491
	LC2 “easy images fast, difficult images slowly”	0.992	0.716	1.375
	LC1 “fast and erroneous”	1.536	1.298	1.817
“How well do you perform in arts education generally?”	LC4 “slow working”	1.057	0.686	1.629
	LC2 “easy images fast, difficult images slowly”	0.591	0.435	0.802
	LC1 “fast and erroneous”	0.721	0.605	0.860

Students were able to correctly identify the required targets virtually without errors for the Exhibition and the Oppermann image (Table 1) and solved the search task in mean durations of about 20–25 s. The Clock and Graffiti image comprised a lot of optical distractors and therefore led to an error rate of at least 11.5% per target. Targets in the Footprint image were much harder to identify with at least one third of all students failing per target. The two more difficult images required on average double the task duration (about 50 s) as compared to the other two images. Remarkably, the third target of each of the four images was the most difficult one for all four tasks. Footprint target 3 was only correctly solved by slightly more than 10% of the students. VL experts, but also novices on average solved the easy images faster than students, with the exception of the Clock and Graffiti image, where VL experts worked longer than students (H1a). Experts were as good or better at identifying targets in comparison to the student sample (H1b). Even though experts found on average one target more than non-experts *M*_Expert_ = 22.76 (*SD* = 1.69), *M*_Non–experts_ = 21.56 (*SD* = 2.65), this was not statistically significant; two-sided Welch *t*(40.781) = 1.91, *p* = 0.063 (H2a). Across all 9 images experts did not differ from non-experts with respect to time on task [*M*_Expert_ = 45.30 s, *M*_Non–experts_ = 45.91 s, *F*(1,48) = 0.022, n.s. (H2b)].

When error patterns over all four images and invested time periods were grouped into latent classes of similar behavior, a latent profile analysis resulted in four distinguishable patterns (see methods section for details justifying the decision for four classes). Latent class 3 (LC3, *n* = 740) more or less represents the same solution pattern (error rates and durations) as the total sample with the exception of Clock and Graffiti, where LC3 performed better than the average. Errors cumulate in the second largest class LC1 (*n* = 238), where students performed reasonably on the Exhibition and Oppermann image, but failed to identify targets over base rates of the Clock and Graffiti and the Footprint image. The reason for this low performance might be given by the high task-performance speed that members of LC1 displayed especially for the more challenging images. The remaining quite small groups (LC2 and LC4) differ mostly with respect to the time invested for solving the search tasks. LC2 (*n* = 56) worked fast on the two easy images (and achieved nearly perfect hit rates), but invested much more time (96 and 125 s) for the more difficult images. By doing so, they were able to achieve hit rates comparable to or better than the “mainstream group” LC3. By contrast, members of LC4 (*n* = 31) represent a group that continuously worked quite slowly (all mean times above 50 s) over all four images, but ending up in error rates not better than the mainstream (see Figure 3).

A multinomial logistic regression model on the solution pattern as represented by class membership explored the potential impact of gender, age, and metacognitive self-perceptions of students in Sample I. Only three variables reached a nominal significance level of *p* < 0.05. Gender and age did not affect solution patterns, neither did the variables “Art is important for me personally,” “When I see a photographed geometric object, I can imagine what it looks like from behind,” “I can remember small details in pictures,” “I like to solve picture puzzles,” “Do you regularly attend an art school or art workshops?”, “Understanding art history and theory.” But the three variables listed in Table 2 had a significant impact on class membership. The global Likelihood Ratio Test for the whole model scored at χ^2^_(__9__)_ = 64.903 (*p* < 0.001) and resulted in a Pseudo-*R*-square of 0.0746. Each of the three regressor variables reached a Wald Chi-square test with *p* < 0.001. Specific effect sizes (Odds Ratio per increasing response category) of the independent variables on the probability of each solution pattern (LC4, LC2, and LC1 compared to the mainstream pattern LC3) are listed in Table 2.

Students claiming to have a photographic memory display a lower probability for belonging to each of the three non-mainstream latent classes at each increased response category. Most pronounced is this effect for LC1 (“fast and erroneous”). Students belonging to this group (on average) proclaim to have a photographic memory to a smaller degree. If students are convinced of their ability to visualize things mentally, then this slightly diminishes their chances to belong to latent classes LC4 and LC2, but increases the probability for membership in LC1 (“fast and erroneous”) by more than 50% per category. This means, that LC1 has a self-perception of high competence in recognizing details in pictures and might therefore work very fast on the respective tasks, but indeed fail to reach the same precision as the other groups. Students’ self-reported high performance in art education is associated with higher chances to belong to the “slow working” group LC4, but considerably lower chances to belong to LC2 or LC1.

From these results it seems clear that interpreting a simple score of correctly solved search tasks does not cover art related visual competence in a meaningful way. A deeper understanding of the cognitive processes during search tasks has to be acquired from additional images and from comparing VL experts to VL non-experts. Results from sample II might contribute to this understanding.

### Eye-Movements in Sample II

Table 3 shows the mean fixation duration and mean number of fixations on each image. VL experts generally show longer fixation durations than non-experts. Experts’ mean fixation durations ranged from 264.83 ms (“Linocut Pattern”) to 317.59 (“Abbey”) and non-experts’ mean fixation duration ranged from 256.26 ms (“Linocut Pattern”) to 308.12 ms (“Abbey”). For most images, experts exhibited more fixations than non-experts. As there is a significant difference in age between both expertise groups [*t*(34.163) = 3.252, *p* < 0.01 with *M*_Experts_ = 34.36, *M*_Non–experts_ = 23.80], a correlation between age and eye movement indicators was calculated for possible confounders. Age and fixation duration exhibited a moderate correlation of *r* = 0.37, *p* < 0.01 and no correlation between age and overall number of fixations was found (*r* = 0.08, n.s).

**TABLE 3:** Mean fixation duration (ms) and number of fixations per image.

Image	Experts (*N* = 25)	*SD*	Non-experts (*N* = 25)	*SD*
**Mean fixation duration (ms)**				
Exhibition	302.35	260.12	287.94	249.74
Oppermann	292.27	230.77	289.26	227.65
Footprints	284.29	195.00	265.24	182.67
Spider net	280.09	228.07	288.71	221.24
Easter	274.69	201.86	258.32	180.26
Abbey	317.59	227.79	308.12	222.22
Clock and Graffiti	297.96	191.01	287.28	197.67
Glasshouse*	313.90	219.25	292.40	201.58
Linocut Pattern*	264.83	148.37	256.26	144.71
**Mean number of fixations**				
Exhibition	59.83	15.76	54.56	19.24
Oppermann	43.83	18.92	40.24	21.19
Footprints	129.46	48.21	154.00	117.97
Spider net	136.67	55.17	104.84	33.48
Easter	117.17	43.13	108.92	60.41
Abbey	136.42	64.07	142.40	47.16
Clock and Graffiti	168.62	92.71	141.04	71.24
Glasshouse*	177.22	107.66	162.48	85.99
Linocut pattern*	298.78	110.99	346.80	205.27

*N = 24 experts due to insufficient data transmission from eye-tracker during one session.

#### HMM Estimates

Table 4 shows the optimal number of hidden states based on HMM by expertise group, i.e., fixation sequences of all experts are used to estimate one HMM for the entire expert group and all fixation sequences of all non-experts for the non-expert group. There is only a small difference in the number of states in the expert group and in the non-expert group (Mean_Experts_ = 8.7 hidden states vs. Mean_Non–Experts_ = 7.8 states) that varies depending on given image.

**TABLE 4:** Optimal* number of hidden states based on HMM by status group.

Image	Expert group (*N* = 25)	Non-expert group (*N* = 25)
Exhibition	9	9
Oppermann	7	8
Footprints	9	6
Spider net	10	7
Easter	7	7
Abbey	8	10
Clock and Graffiti	8	8
Glasshouse**	10	9
Linocut pattern**	11	7

*As indicated by Bayesian Information Criterion (BIC), **N = 24 Experts due to insufficient data transmission from eye-tracker during one session.

**FIGURE 5:**
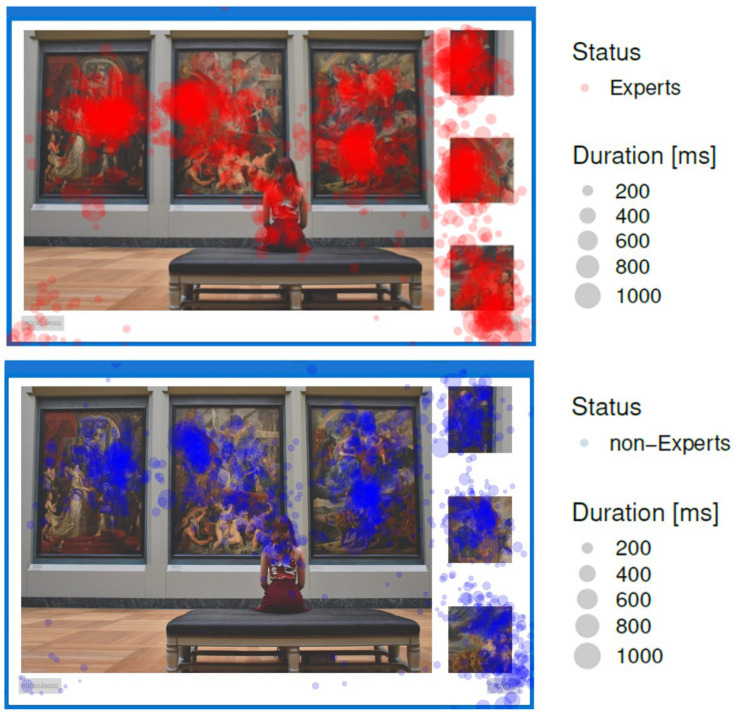
Heatmap of fixation points of experts (red) and non-experts (blue) on image Exhibition. Each circle represents a fixation. Size of circles represents fixation duration.

A traditional Heatmap (Bojko, 2009) of fixations and fixation durations of Experts and non-Experts is given on the image Exhibition (Figure 5). As can be seen, fixations tend to cluster around prime and target image regions. Also some fixated areas seem to overlap each other. What about the fixations in between, i.e., to WS? If we want to classify each fixation to their respective underlying image region, we can use the HMM to infer the most probable state for each fixation. We also take the sequence of fixations, as a transition probability matrix into account while modeling [in contrast to other classification methods like k-means clustering (Steinley, 2006)].

**FIGURE 6:**
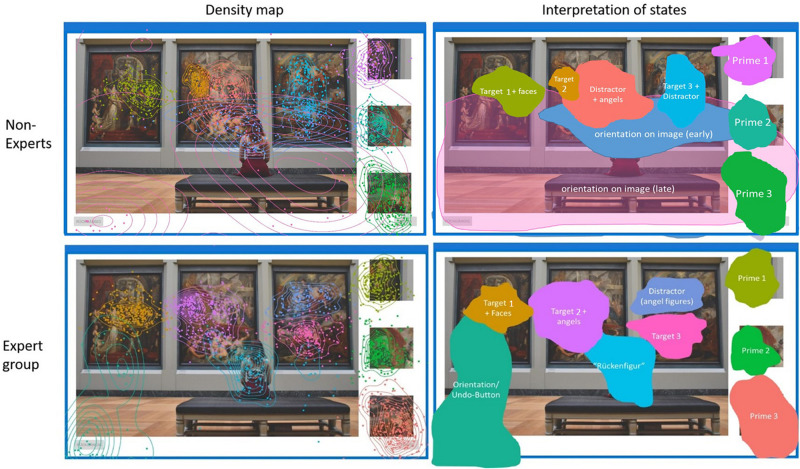
HMM density map of hidden states based on the fixation sequence for each group **(Left)** and assigned semantic interpretation of the hidden states **(Right)**. States are colored for better differentiation. Each hidden state either represents a prime, target or distractor region in WS. The nine states of the non-expert group are: (1) Prime 1, (2) Prime 2, (3) Prime 3, (4) Target 1, (5) Target 2 + faces, (6) Target 3 + Distractor, (7) Distractor + angels, and (8, 9) as two wide orientation states across the image. The nine states of the expert group are: (1) Prime 1, (2) Prime 2, (3) Prime 3, (4) Target 1 + angles, (5) Target 2 + faces, (6) Target 3, (7) angel figures as distractor, (8) “Rückenfigur,” (9) orientation/undo-buttons. See Supplementary Material for all nine images.

As each HMM is based on spatial coordinates of fixation points and their sequence, hidden states can be analogously visualized as 2D topological areas on the image incorporating additional information. Figure 6 represents hidden areas for the non-expert group and expert group on image Exhibition (see E-Appendix for all nine images). Every hidden area was assigned a different label, either belonging to a prime, target or distractor area (part of WS). Interestingly, the novice group includes a broader orientation area which spans across the image. We can differentiate this clearly now, as each fixation is matched to their most probable state over time, allowing for spatially overlapping states differentiated additionally through the transition probability matrix. By contrast, the expert group clearly defines a figure of a woman in front of the museum paintings, resembling the traditional motif of a “Rückenfigur.”

**FIGURE 7:**
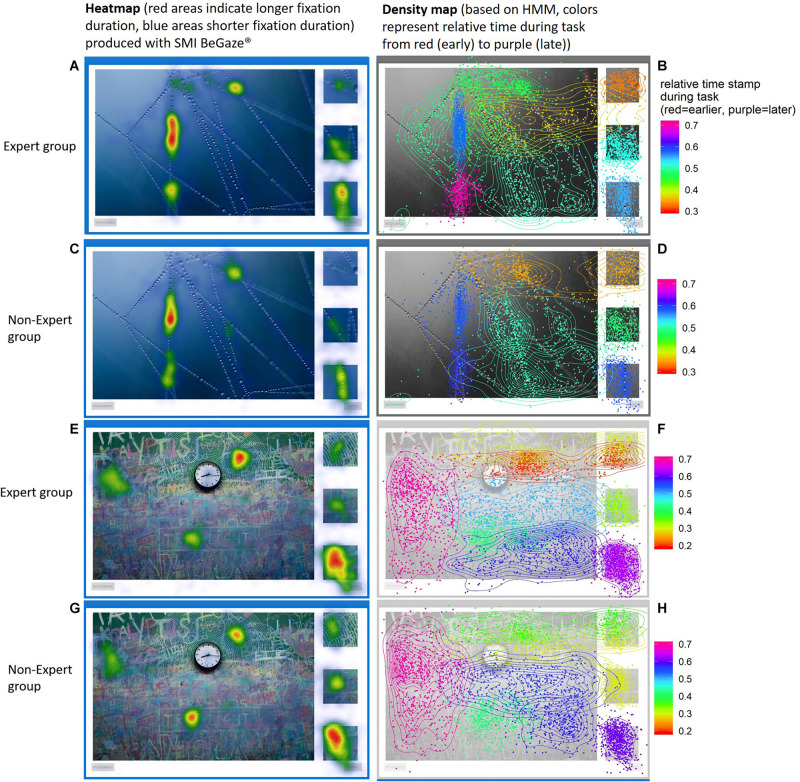
Heatmaps **(A,C,E,G)** vs. density maps **(B,D,F,H)** on images (“Spider Net” and “Clock and Graffiti”) for the expert group and non-expert group. The Heatmaps (left) disregard fixation points that are scattered outside of “hot spots” because on a global scale they do not carry much information. Image underlying density maps are masked out to make colors more visible. The HMM density maps (right) shows how every fixation point is connected to their most probable state throughout the image (color indicates differences in relative time of attendance). Some hidden states span over multiple areas or overlap each other that are still differentiable through time. We can also see how local differences in density differ within each state.

Figure 7 compares another conventional Heatmap (produced by SMI BeGaze) and a density map based on the HMM. Regions attracting attention during different phases of the visual search become visible through HMM states, otherwise they are overlooked in conventional Heatmaps. For example prime 1 (top right corner) is hardly evident by the Heatmap. However, the HMM density map shows us how prime 1 is connected to the target region (at the top) and incorporated into a common state. In this case, experts as well as non-experts seem to fixate two pre-defined AOIs in rapid succession to form a single HMM state. Both examples given in Figures 6, 7 show how regions not previously identified (in WS) that attract participants’ attention are uncovered with the use of HMMs. Figure 7 additionally shows how overlapping hidden states differ in their relative time (normalized to each subject’s individual working time).

**FIGURE 8:**
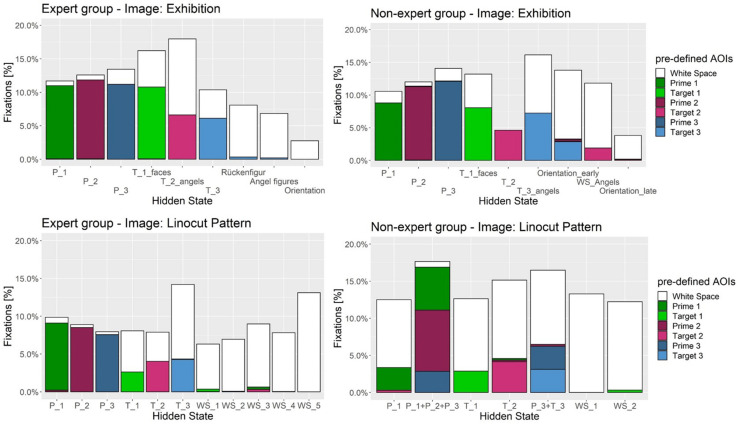
Distribution of fixations and percentage overlap on pre-defined AOI and hidden states for the expert **(Left)** and non-expert **(Right)** group. Each color represents either a target or prime region on the image. White is undefined White Space (WS) area. In most cases prime regions are represented by a single hidden state; e.g., states for target and prime regions in the expert group on image Exhibition. Target regions are usually encompassed by WS area, as they need to be found inside the image. Frequent transitioning between multiple pre-defined AOI may integrate into one hidden state; for example in the non-expert group on image “Linocut Pattern” (bottom right) one hidden state overlaps with P_1, P_2, and P_3.

How good does each hidden state represent pre-defined AOIs? Figure 8 displays the overlap of hidden states with pre-defined AOIs. There is a clear connection between the hidden states and the pre-defined AOIs. In most cases one hidden state is directly associated with one prime or target AOI, indicating a meaningful distribution of hidden areas on the image. The more “white” a hidden state encompasses the more it includes undefined WS region on the image. Usually the target AOIs include more WS. Some hidden states in WS can be defined as distractor areas that draw attention during the search. When a hidden area covers multiple pre-defined AOIs (see Figure 8 on image “Linocut Pattern” below, e.g., in state 2 for non-experts) they combine into a single state. This may happen due to frequent transitioning between two pre-defined AOIs. We can see how the states are not randomly distributed over the image, but are closely related to the visual search task (primes and targets).

**FIGURE 9:**
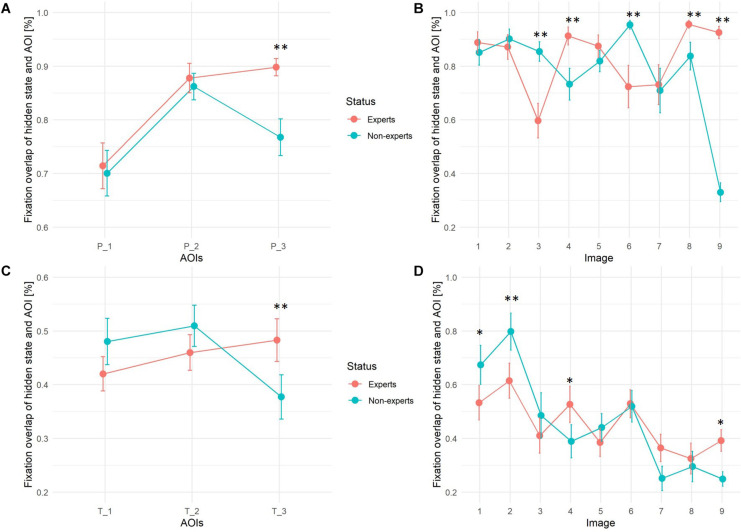
Fixation overlap between pre-defined AOIs and best fitting hidden state (state with highest fixation overlap) for expert and non-expert group. Upper panels **(A,B)** show overlap between prime regions, lower panels **(C,D)** between target regions. Images: 1 = Exhibition, 2 = Oppermann, 3 = Footprints, 4 = Spider Net, 5 = Easter, 6 = Abbey, 7 = Clock and Graffiti, 8 = Glasshouse, 9 = Linocut Pattern. ^∗∗^*p* < 0.001, ^∗^*p* < 0.05.

In order to determine differences in precision between experts and non-experts (H3), hidden states in each HMM were assigned to 3 primes and 3 target AOIs according to their best possible fit; each pre-defined AOI to the HMM-state with the most fixation overlap, i.e., the percentage of fixations of the corresponding HMM that fell into the predefined AOI region, (see Figure 8). A two-way, repeated measures (three primes and three targets as within factor 1, nine images as within factor 2) ANOVA with one between-subjects factor (VL-experts vs. non-experts) was estimated using these percentages as dependent variable measuring the precision of the fixations, one ANOVA model for primes, one for targets. No main effect for experts vs. non-experts could be seen in either of the models: though the global *F*-test for primes pointed at differences [*F*_primes_(1,48) = 13.25], the global *F*-test for targets [*F*_targets_(1,48) = 3.96] fell short to reach significance and no clear direction of differences was visible (see Figure 9). For both primes and targets there was a significant main effect for images [*F*_primes_(8,41) = 182.77; *p* < 0.001; *F*_targets_(8,41) = 67.33; *p* < 0.001], and for the three AOIs [*F*_primes_(2,47) = 105.38; *p* < 0.001; *F*_targets_(2,47) = 8.84; *p* = 0.0006].

Both within-subjects effects interacted with the status of experts vs. non-experts in the following way: the third prime and target AOI (P_3 and T_3) on all 9 images was on average fixated by experts with a significantly higher precision than by non-experts (see Figure 9). Non-experts on P_3 and T_3 more frequently switched between prime and target and WS regions to reassure that they recognized the correct region on the image, whereas VL-experts once they had memorized the third prime (coverage rate nearly 90% vs. 75%), VL-experts were able to search the corresponding target quite efficiently (coverage rate nearly 50% as compared to only 37% in non-experts). With regard to differences between images, VL-experts reached a significant higher precision in fixating the primes on the “Spider Net,” “Glasshouse,” and “Linocut Pattern” image, and in fixating the targets of the “Spider Net” and “Linocut Pattern” image. Non-experts on the other hand more precisely fixated the primes of the “Footprints” and “Abbey” image indicating that they spent more fixations within the prime regions and reached higher precision in fixating target regions in image “Exhibition” and “Oppermann.”

## Discussion

### Student Solution Patterns

The majority of students (*n* = 740) took their time to solve the more difficult items “Footprints” and “Clock and Graffiti”. Most of these “mainstream” students claimed to have a ‘photographic memory’ to a higher degree than other student groups. Overconfidence in student’s ability to “visualize images mentally” increases the probability of them rushing through the tasks and making more mistakes. Visualizing images mentally can go beyond details in 2-dimensional images and therefore not be helpful for the search task. A “photographic memory” much more encompasses the necessary skill to attentively focus on details in artwork. On the contrary, high self-reported capacity in art education leads students to take it slower on all tasks but does not necessarily enable students to outperform other student groups. Art teachers therefore may be interested not only in students engagement in art class but also be inclined to know about visual memory (“photographic memory”) skills. These results can be first clues for finding student groups that need more help in engaging and analyzing artwork or to learn when to invest more time in a visual task.

VL experts could solve three of the common four images faster than students and in a more homogeneous manner (less variance of solution time) (H1b) and at equal or even superior chances for a correct solution (H1a). Only for the “Clock and Graffiti” image did VL experts take longer than students, resulting in a nearly perfect solution probability that was not reached by students.

### Expertise Differences

Did VL experts outperform students? This cannot be deduced from mere test solutions, but requires data on the solution process as well. Because the pathway to understanding cognitive processes is revealed by the analyses of eye movement data, we recorded VL experts’ oculomotor behavior while working on the tasks. Clearly this measurement could not be performed in a large classroom survey. Therefore, VL experts were compared to non-experts in Experiment II.

Visual literacy experts found as many targets and were as fast as non-experts (H2a, H2b) but differed in the way they found target regions. HMM analysis revealed that experts were able to divide seven of the nine images into the same number of or more areas (hidden states) than novices. Aesthetic interpretation of hidden states suggest that WS does not cover a homogeneous region of “non-attraction” but has meaning and “Gestalt” that goes beyond the gist of the scene and governs clues important for the scene composition. The idea that a sequence of hidden states represents cognitive processes is further emphasized by the visualization of the hidden states of experts with density maps, which revealed semantically meaningful regions on the image. Dissecting the image into these additional regions might help VL experts in understanding the scene composition, best illustrated by specific symbolic objects (angel-figures) or artistic compositions (“Rückenfigur”). These regions might be more salient for VL-experts. Given experts knowledge about image composition and arrangement, experts may be able to “find their way” through the pictures differently. Results hint at fewer hidden states for the non-expert group and a wider spread of fixations across WS compared to experts. This is in accordance with the findings of Koide et al. (2015) stating that experts regarding abstract paintings tend to not only focus their attention on salient regions (for a visual search task, the target and prime regions) but were also able to direct their attention to areas that are disregarded by novices. One possible explanation would stress the role of working memory as the psychological correlate of the underlying cognitive processes during the respective spatiotemporal HMM state. Thus, important information for the image arrangement is processed while fixating a certain region of the image during a given time period (Irwin, 2004).

To assess the precision of expert and non-experts search strategy we assigned hidden states to each pre-defined AOI with the highest fixation overlap. ANOVA revealed a significant interaction of fixation overlap (of hidden state and AOI) with expertise, the image and the pre-defined AOIs. Precision is higher for the expert group on the third prime and target regions (H3). Non-experts show more fixations in previously undefined WS when they look at the third prime and search for the third target region, leading to more fixations outside pre-defined AOIs within the corresponding hidden state, therefore focusing with less precision than the expert group.

Recent evidence has shown that aesthetically appreciated images lead to enhanced perceptual processing (Sarasso et al., 2020). VL experts would have therefore benefited from the artwork stimuli as it might have improved the engagement and encouraged a deeper commitment into “analyzing” (Wagner and Schönau, 2016) the image thoroughly. It can also be argued that visual working memory (Olivers et al., 2011; Bahle et al., 2019) of experts form an enhanced representation or mental model of the images.

### Hidden Markov Models in Visual Search

Each HMM state in Sample II represents a location on the image participants fixated while inspecting that area during the search. In general, there is a good coincidence between pre-defined AOIs (primes and targets) and the data driven hidden states. WS mostly comprises several distinguishable hidden areas either representing semantically meaningful attractors (e.g., people’s faces or control buttons) or distractor regions that had to be excluded (e.g., different angel-figures or different parts of the spider net).

Hidden Markov models also allows for an aggregated comparison between fixation sequences. Usually comparing multiple scanpaths between subjects is a complex challenge. E.g., what threshold values should be used to define a starting/landing point for fixations on important image areas? The HMMs based on group level allows for a description of fixation sequences as hidden states on the image, as each fixation point was classified to their most probable hidden region over time.

Hidden Markov models have not been extensively used in visual search tasks. However, the method presented here, can be of great use to investigate complex search processes that can go beyond the visual search task presented in this study (e.g., natural scenes, virtual reality, and real-world searches) in which classical aggregate statistics may fall short. Subtle differences in viewing behavior can be more clearly defined. The hidden states estimated by the HMM can be interpreted as data-driven AOIs. Instead of defining AOIs by arbitrary thresholds, we can include subject’s fixations that lie outside the pre-defined AOIs in WS to be included to any data-driven AOI (hidden state) based on the estimated probability.

The HMM presented here are not exhaustive for eye movement analysis for expertise research and can be expanded upon (e.g., additional variables are conceivable as basis for model formulation such as the length of saccades or individual fixation durations to assess selective attention allocation). Further research is needed in respect to VL skills in the domain of arts, as the literature is dominated by studies in sports and medicine and eye movement differences are heterogeneous across expertise domains (Brams et al., 2019).

### Limitations

A few limitations have to be mentioned. First, the images were not tested for low-level saliency (Foulsham, 2019). Our focus was on expert vs. non-expert search strategies in identifying targets from prime regions. Different levels of saliency could interfere with the results presented here (Loftus and Mackworth, 1978). Thus, further studies could vary the number of visual salient features systematically. That way cognitive states prone to bottom-up mechanisms could be differentiated from top-down search strategies guided by expertise and/or working memory.

Another concern for the generalizability of our results is the lack of time constraints combined with a motor coordination problem in identifying the targets. Deviating from earlier studies, this might lead to a varying number of data points per trial. The visual search task was only finished when the participant indicated to have found all three target regions. This led to a wide variance in individual search times untypical for other target search experiments that only last a few seconds (Coutrot et al., 2018). It remains uncertain how many fixations are efficient for HMM for eye movements. Depending on the time constraints and task at hand, different number of fixation points might be necessary. Coutrot et al. (2018) argue that images should contain various regions of interest to capture systematic patterns of exploration behavior. This can be achieved by pre-defined AOI (as in our study) or by using images with different salient areas that draw participants attention.

Lastly, even though the number of participants was above average for eye tracking studies in expertise research (Gegenfurtner et al., 2011), sample size might still have been too small to find all differences between VL experts and non-experts, especially as effects of VL on visual search behavior seem to be more subtle than proposed by art education research. As the present results are exploratory, further research is needed to confirm these observed differences. There was also a mean age difference of 10 years between each group. However, as the number of fixations was not correlated with age, we would argue that differences in HMMs are not primarily due to age differences. A few seconds of eye movement data used to define fixation sequences was sufficient to model clearly distinguishable hidden states on a group level. This is promising for future use cases with limited sample size and more obvious differences in eye-movement behavior [e.g., in patients with visuospatial neglect (Cox and Aimola Davies, 2020)].

### Conclusion and Future Outlook

This study investigated VL expert and non-expert visual search behavior. The expert group revealed a more detailed search strategy, indicated by a higher number of hidden states and higher precision for looking at the last prime and searching for the last target. Specific image parts, previously not taken into account by pre-defined AOI were outlined in greater clarity among VL experts. Non-experts on the other hand, focused on broader and thus fuzzier image areas during visual search. For the purpose of constructing a VL assessment test battery, selecting more items of intermediate or greater difficulty, including even more realistic and stylistic image compositions, is advised because students displayed a rather skillful ability to perform visual search tasks.

From a methodological view point, the statistical methods used could introduce a new perspective on modeling expertise-related differences in eye movements. Future studies could investigate the link between topological HMM states and “cognitive” hidden states incorporating more variables such as fixation duration or saccadic length into the models. The same idea was followed by van der Lans et al. (2008), who found an association between a 2-state cognitive HMM based on local and global search strategies. Deviating from our art oriented approach in choosing visual stimuli, they used a saliency map based on low-level perceptual features and the scene’s organization to explain their results. Other recent approaches measured oculomotor behavior while switching between hidden cognitive states during a decision task (Chuk et al., 2019). In an educational context, not necessarily restricted to art education, HMMs states could be helpful to describe how much students are “involved” in the given tasks.

## Data Availability Statement

The raw data supporting the conclusions of this article will be made available by the authors, without undue reservation.

## Ethics Statement

The studies involving human participants were reviewed and approved by Ethics Committee of Research of the Leibniz Institute for Research and Information in Education, Frankfurt am Main. Written informed consent to participate in this study was provided by the participants’ legal guardian/next of kin.

## Author Contributions

MG, UF, and KR designed the study. MT and EW selected and prepared the stimuli. MT conducted the field work. MT and UF designed and performed the statistical analysis. EW contributed aesthetic theory to the interpretation of statistical results. MT, MG, KR, and UF prepared the manuscript. All authors reviewed the manuscript.

## Conflict of Interest

The authors declare that the research was conducted in the absence of any commercial or financial relationships that could be construed as a potential conflict of interest.
