# Supplementary material for: How Do Art Skills Influence Visual Search? – Eye Movements Analyzed With Hidden Markov Models
Source: Front Psychol. 2021 Jan 28;12:594248. doi: 10.3389/fpsyg.2021.594248 (PMC7875865; doi:10.3389/fpsyg.2021.594248)

Image title | **HMM Density map**

Non-expert group

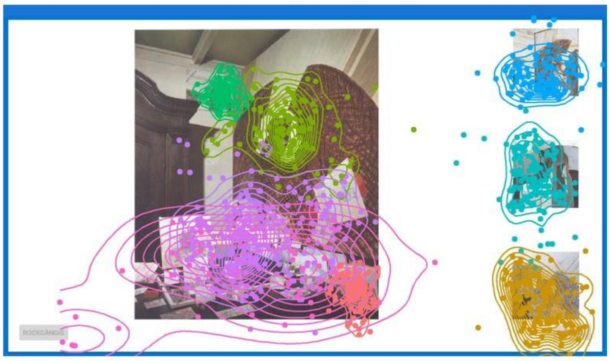

Oppermann

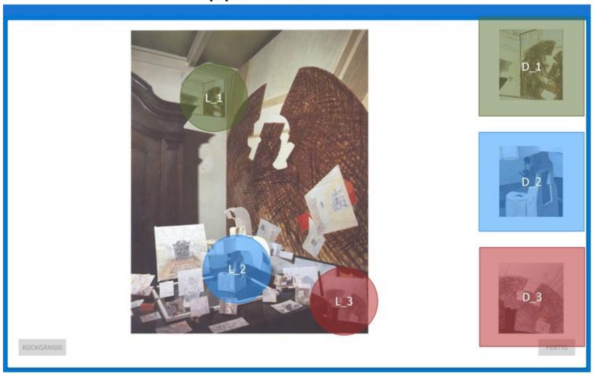

Expert group

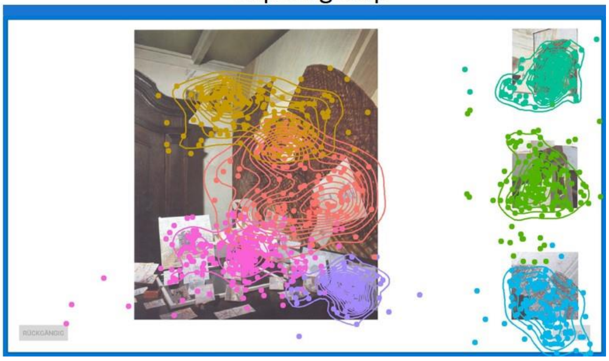

Non-expert group

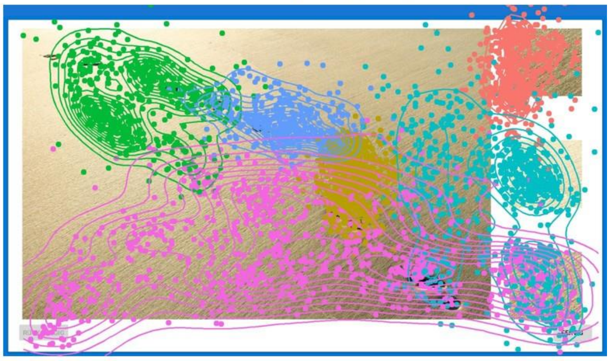

Footprints

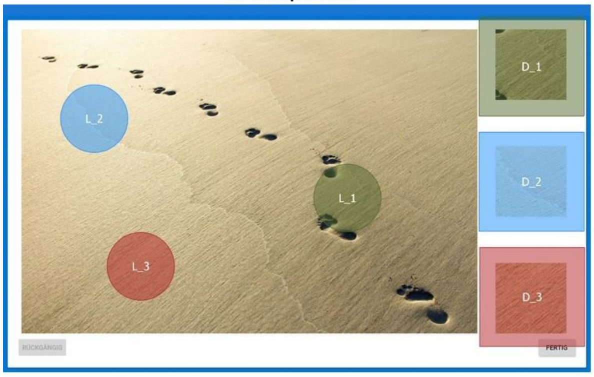

Expert group

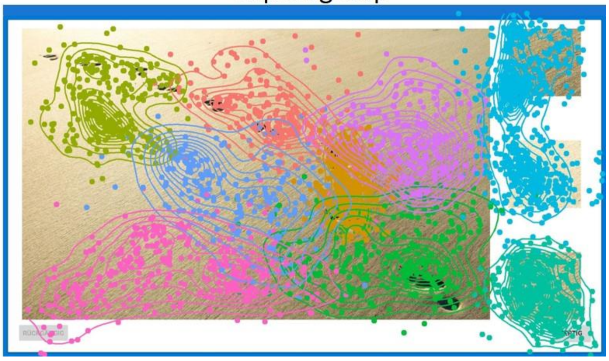

Spider Net

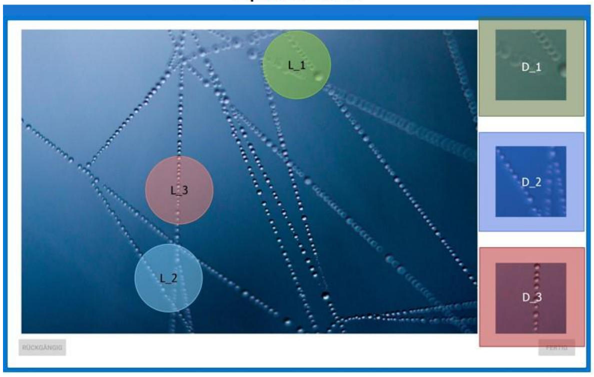

Non-expert group

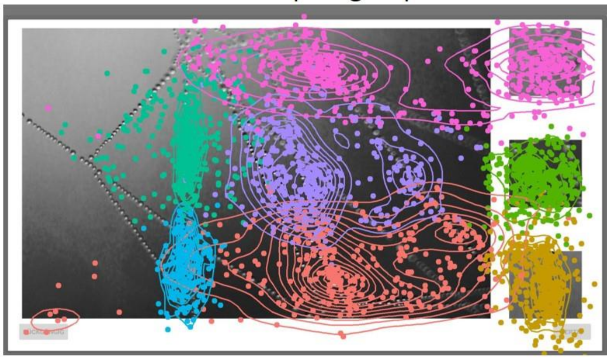

Expert group

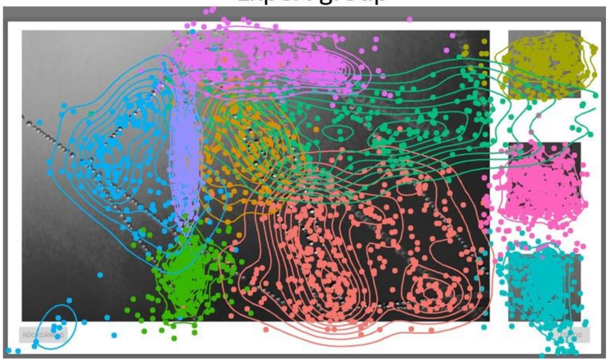

Easter

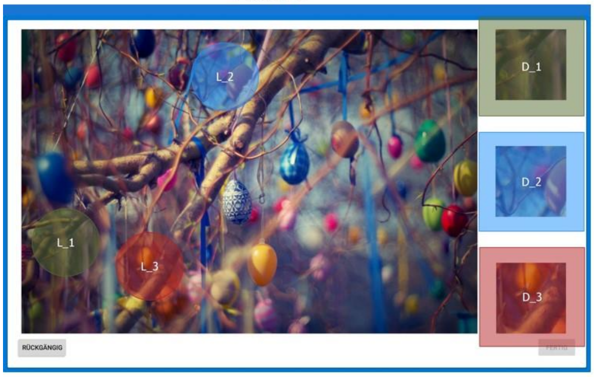

Non-expert group

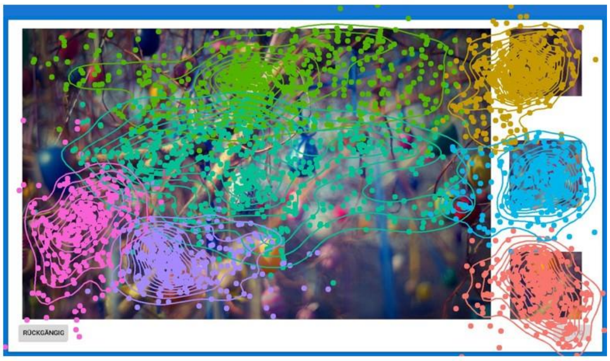

Expert group

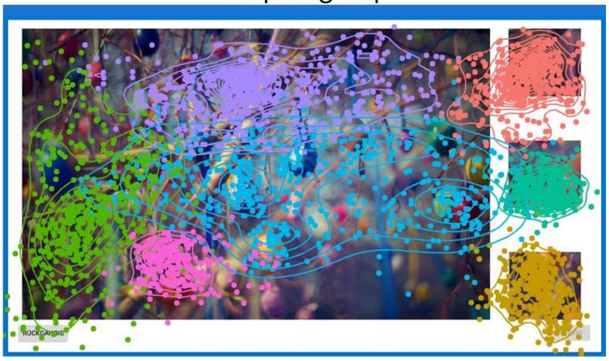

Abbey

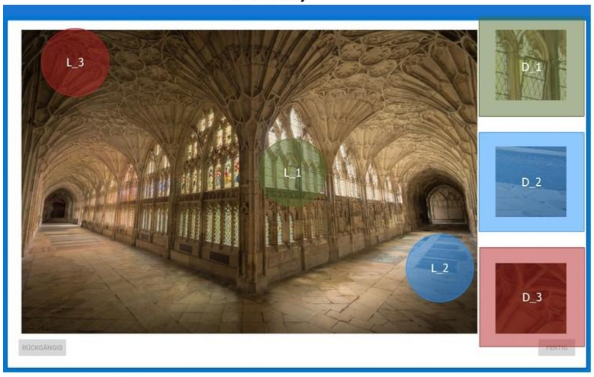

Non-expert group

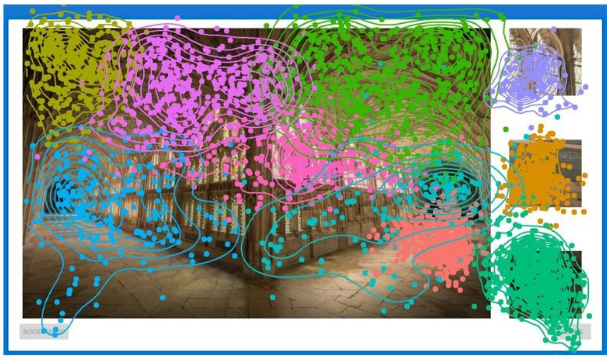

Expert group

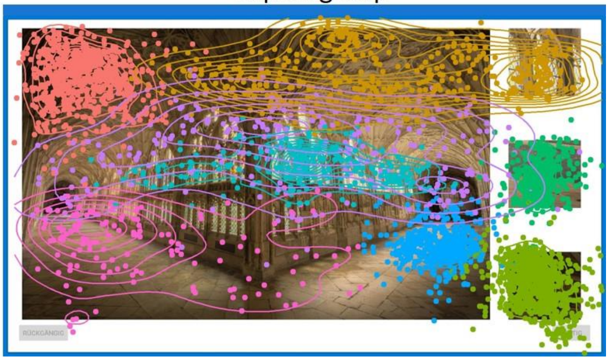

Clock & Graffiti

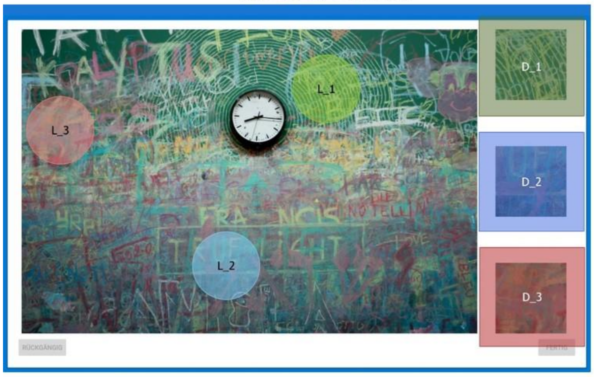

Non-expert group

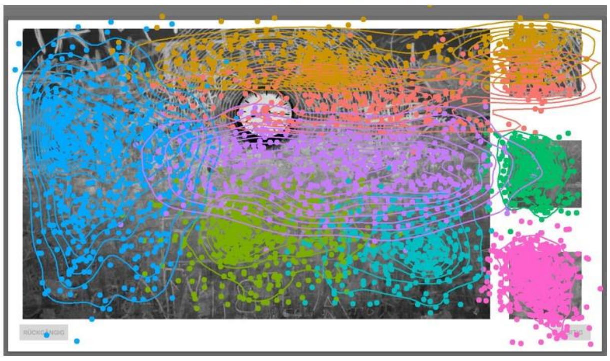

Expert group

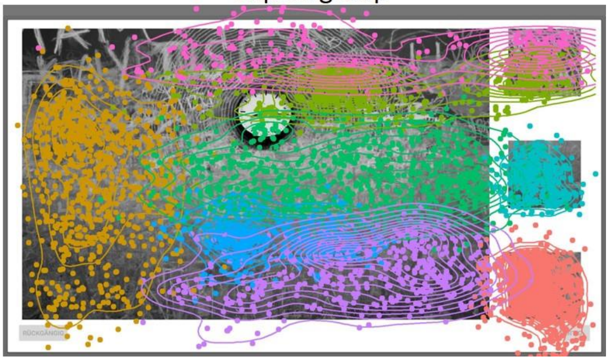

Glasshouse

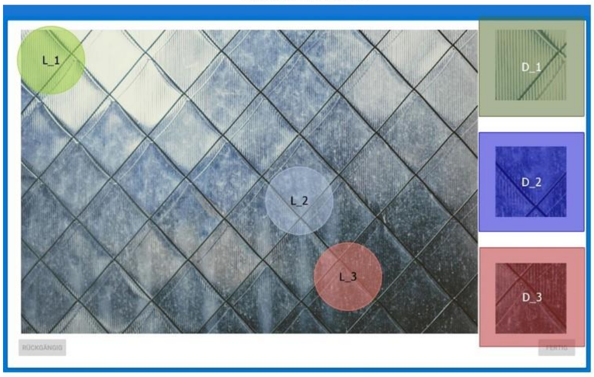

Non-expert group

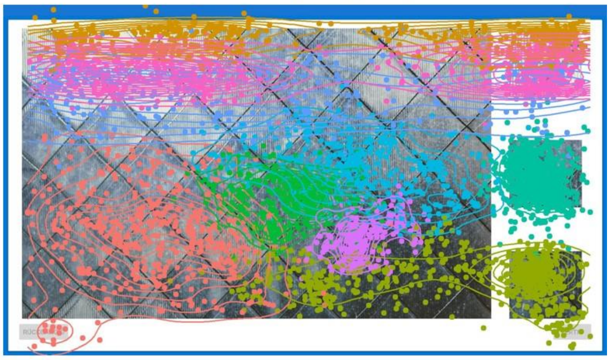

Expert group

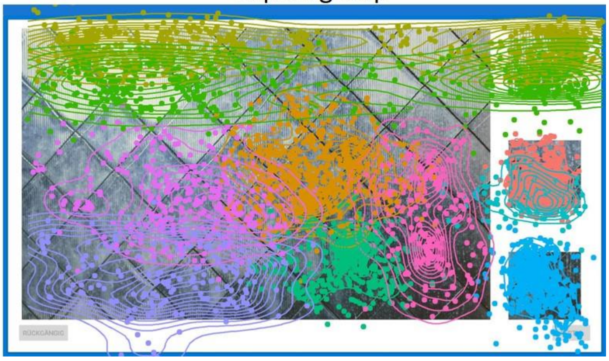

Linocut Pattern

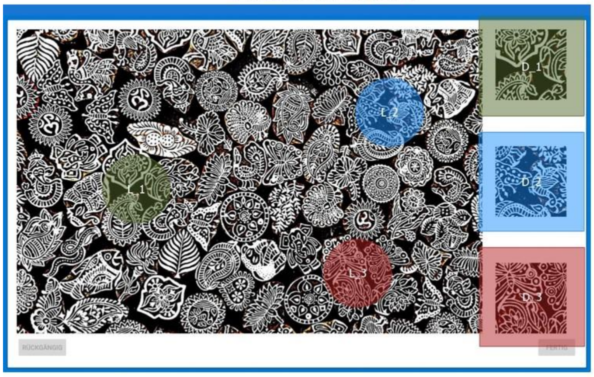

Non-expert group

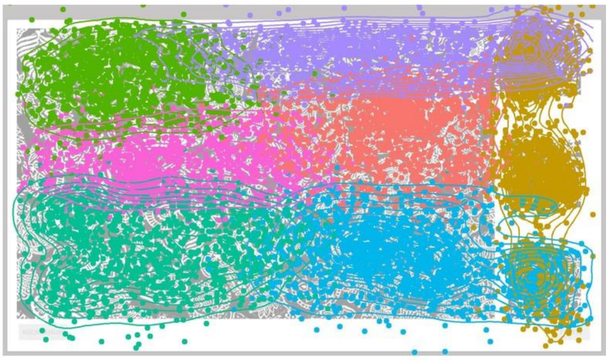

Expert group

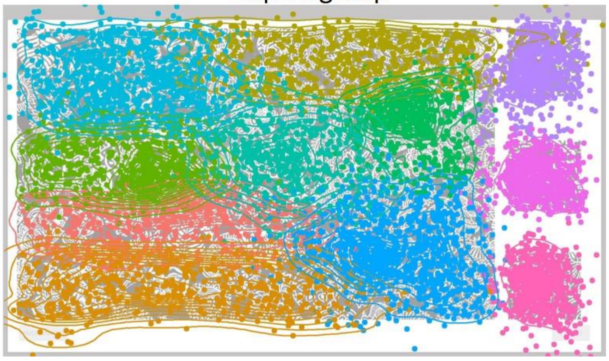

Supplement: Supplementary file 1 [file Data_Sheet_1.PDF]
